# Evaluation of Antidepressant-like Effect of *Citrus Maxima *Leaves in Animal Models of Depression

**Published:** 2011

**Authors:** Vikram H Potdar, Swati J Kibile

**Affiliations:** 1 *Department of Pharmacology, Tatyasaheb Kore College of Pharmacy, Warananagar, Tal: Panhala, Dist: Kolhapur, Maharashtra, India*; 2 *Tatyasaheb Kore College of Pharmacy, Warananagar, Tal: Panhala, Dist: Kolhapur, Maharashtra, India*

**Keywords:** Antidepressant-like effect, Citrus maxima, Forced swimming test, Tail suspension test

## Abstract

**Objective(s):**

This study planned to assess antidepressant like activity of aqueous extract from leaves of *Citrus maxima* Merr. (Rutaceae).

**Materials and Methods:**

Boiling was used for aqueous extraction. Acute toxicity study was performed in mice. Antidepressant activity was studied using locomotor activity test, modified forced swimming test (FST) and tail suspension test (TST). Three doses 100, 200 and 300 mg/kg of aqueous extract of leaves were selected for testing. Fluoxetine (20 mg/kg, i.p.) and imipramine (30 mg/kg, i.p.) were used as the standard drugs.

**Results:**

Aqueous extract of *Citrus maxima* leaves significantly reduced immobility time in both TST and FST. In locomotor activity testing it showed psychostimulant effect. Extract increased the climbing behavior in FST, which is similar to effect observed with imipramine.

**Conclusion:**

The results of this study suggest that antidepressant like effect of *Citrus maxima* seems to be mediated by an increase in norepinephrine level in synapses.

## Introduction

Depression is a serious mood disorder that afflicts several millions of the world population. Furthermore, the World Health Organization revealed that depression is the fourth leading cause of disability worldwide, exceeded by lower respiratory infections, perinatal conditions and HIV/AIDS (1). Approximately, two third of depressed patients experience suicide thoughts and 10-15% of them attempt suicide (2). The main symptoms of depression are due to functional deficiency in the levels of monoaminergic transmitters noradrenalin, 5- hydroxytriptamine and dopamine in the brain (3). Drugs that increase the level of these neurotransmitters in the CNS show antidepressant activity (2).The major antidepressant therapies aim for an enhancement in the transmitters levels in the neurons and thus normalize the neurotransmission (4).

Many of the currently available antidepressant drugs have proven to be effective but they are burdened with some disadvantages such as various adverse effects, problematic interactions and relatively low response (5). In addition, it is also reported that only two out of three patients respond to any given treatment and, of these, one would probably have responded to placebo alone (6). On the other hand, drugs obtained from natural sources have good efficacy, least risk and low side effects profile. Recently, the search for novel pharmacotherapy from medicinal plants for psychiatric illnesses has progressed significantly. Therefore, herbal therapies should be considered as alternative or complementary medicines (7).

*Citrus maxima* Merr. (Rutaceae) is known as pummelo. The pummelo tree is normally 16 to 50 ft tall, with a somewhat crooked trunk 10-30 cm thick and low, irregular branches, large leaves with winged petioles. It is commonly known as ‘papanasa’ in . Literature survey revealed the presence of β-sitisterol, acridone alkaloids in root and bark of *C. maxima*. The essential oil from the leaves and unripe fruits contain limonin, nerolol, nerolyl acetate and geraniol. It has been used in indigenous system of medicine as sedative in nervous affections, convulsive cough and in the treatment of hemorrhagic diseases and epilepsy (8). 

As per traditional claim, the plant possess an anti-convulsant activity, but the results (data not shown) which we got in our laboratory by performing practical on animals with different doses of aqueous extract of *C. maxima* leaves, were found to be negative for anti-convulsant activity. Rather the results which were obtained from the effect of same doses of extract on animals in locomotor activity testing indicate that this extract has CNS stimulant activity. These findings promoted us to study its antidepressant activity. Thus the aim of the present study was to evaluate possible antidepressant effect of aqueous extract of* C. maxima* leaves in various animal depression models.

## Materials and Methods

***Animals***

Swiss albino mice weighing 25-30 g, of either sex were procured from the central animal facility of the Institute and maintained under the standard conditions: room temperature (25±3) °C, humidity 45%–55%, 12 /12 hr-light/dark cycle. They were fed with commercially available mouse normal pellet diet and water was allowed *ad libitum*. The experimental protocols were approved by the Institutional Animal Ethics Committee of College.

***Plant material and preparation of extract***

The leaves of *C. maxima* were collected and authenticated by botanist Dr Ashok S Nigwekar, Bhogavati mahavidyalaya, . Leaves were washed thoroughly with water and air dried in shade at room temperature. For aqueous extract, 250 g powder of leaves was boiled in 1 liter of distilled water for 30 min. Subsequently, the mixture was filtered using Whatman filter paper. The filtrate was concentrated over the vapor of the water bath and dried under vacuum. The percentage yield of extract was 9.76%. 

***Acute toxicity study***

Acute toxicity study was performed in mice. The extracts were administered orally at doses of 175, 550, 2000 mg/kg. Animals were observed for signs of toxicity, continuously for 2 hr, and for mortality up to 48 hr, after oral administration of different doses of extract (9).

***Experimental protocol***

*Grouping and drug treatment*

The animals were grouped into 18 different groups, each containing 6 animals, according to different tests of antidepressant activity as follows:

Group 1, 2, 3: Distilled Water 10 ml/kg (p.o.) [Control for locomotor activity testing, forced swimming test (FST) and tail suspension test (TST) respectively.]

Group 4, 5, 6: Fluoxetine 20 mg/kg (i.p.) [Standard for Locomotor Activity testing, FST and TST respectively.]

Group 7, 8, 9: Imipramine 30 mg/kg (i.p.) [Standard for Locomotor Activity testing, FST and TST respectively.]

Group10, 11, 12: Aqueous extract of* C. maxima* leaves 100, 200, 300 mg/kg (p.o.) respectively.

Group13, 14, 15: Aqueous extract of *C. maxima* leaves 100, 200, 300 mg/kg (p.o.) respectively.

Group16, 17, 18: Aqueous extract of* C. maxima* leaves 100, 200, 300 mg/kg (p.o.) respectively.

Group 10-12, 13-15 and16-18 served as test group for locomotor activity testing, FST and TST respectively. Fluoxetine and imipramine (reference standard drugs) were dissolved in distilled water and administered via i.p. route half an hour before the each test. Test groups were treated with aqueous extract p.o. one hour prior to the test.

***Antidepressant activity***

*Locomotor activity*

The locomotor activity was assessed on naïve pretreated mice using an actophotometer. Actophotometer operated on photoelectric cells which were connected in circuit with a counter. When the beam of light falling on the photocell was cut off by the animal, a count was recorded. These cutoffs were counted for a period of 10 min and the figure was taken as a measure of the locomotor activity of the animal (10).

*Modified forced swimming test*

Antidepressant activity of plant extract was assessed using modified Porsolt test (7). Mice were placed individually in a transparent glass cylinder (12 cm in diameter, height 25 cm), which was filled with water to a height of 15 cm. Two swim sessions were conducted. An initial 15-min pre-test followed 24 hr later by a 6 min test. In the pre test session, the mice which have not yet treated were forced to swim in a glass cylinder for 15 min. In the second session, each mouse received a respective dose of sample 1 hour prior to test, and placed in the cylinders again for 6 min. The following behaviors were recorded during the last 4 min (2).

1. Immobility: floating in water without swimming.

2. Swimming: active movements of extremities and circling in the container.

3. Climbing: active movements of forelimbs on the container wall.

*Tail suspension test*

The tail suspension test (TST) was performed according to the method described by Steru *et al* (11). The principle of this test is that suspending mice suspended upside down leads to characteristic behavior immobility which resembles to human depression. After the administration of respective sample, mice were suspended on the edge of the table 50 cm above the floor by adhesive tape placed approximately 1 cm from the tip of the tail. Immobility duration was recorded for the last 4 min during 6 min period. Mice were considered immobile when they hanged passively and completely motionless.

***Statistical analysis***

All values are expressed as mean±SEM. Data was analyzed using a statistical package (InStat software, ). One-way analysis of variance (ANOVA) followed by Tukey’s multiple comparison test were used. In all the tests, the criterion for statistical significance was *P*< 0.05.

## Results

***Antidepressant activity***

*Locomotor activity in mice*

Between the different doses of *C. maxima* leaves extract used; only the dose of 300 mg/kg has shown significant increase in locomotor activity (*P< *0.001). No sedative effects were observed at any of the doses tested. In contrast, imipramine significantly decreased activity counts (*P<* 0.05), suggesting a sedative effect (Table 1). 

*Effect of* *C. maxima leaves extract* *on immobility time in TST in mice*

As show in Table 2, the results indicate that animals pretreated with aqueous extract of* C. maxima *leaves reduced the total immobility time in TST in mice. The obtained results were found to be statistically significant (*P< *0.05, *P< *0.001 for doses of 200 and 300 mg/kg respectively). 

*Effect of C. maxima leaves extract on immobility time, swimming and climbing in modified FST in mice*

**Figure 1:**
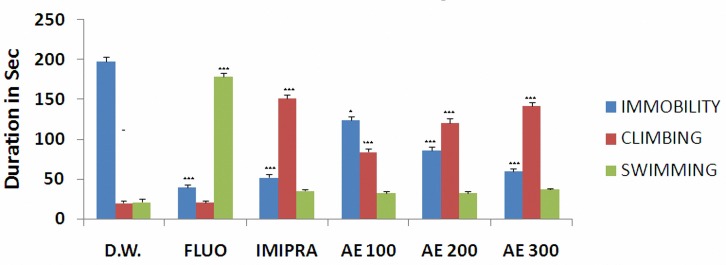
Effect of *Citrus maxima* leaves extract (AE 100-300 mg/kg p.o.) on immobility time, swimming and climbing in modified forced swimming test in mice. Each bar represents mean response from 6 mice±SEM (* and *** indicate *P*  0.05 and *P*  0.001, respectively). [D.W.-Distilled Water, FLUO-Fluoxetine, IMIPRA-Imipramine, AE-Aqueous Extract].

Single administration of fluoxetine and imipramine significantly decreased the immobility time (*P*< 0.001) as shown in Figure 1. Administration of different doses of aqueous extract of *C. maxima* leaves significantly reduced the immobility time in dose dependent manner (*P*< 0.001). The results also showed that accompanied with the decrease in immobility time; extract increased climbing time without significant change in swimming time (*P* 0.05), similar to the effect of imipramine. The reductions of the immobility time were 37%, 56.56% and 69.69% for the extract at 100, 200 and 300 mg/kg, respectively.

**Table 1.** Effect of aqueous extract of *Citrus maxima* leaves on locomotor activity in mice.

Group No.	Treatment	Dose (kg^-1^)	Activity counts (s)
1.	Distilled Water	10 ml	196.5±6.12
4.	Fluoxetine	20 mg	308.66±8.22^***^
7.	Imipramine	30 mg	107.5±5.83^*^
10.	Aqueous extract of *Citrus maxima* leaves	100 mg	212.83±2.86
11.	Aqueous extract of *Citrus maxima* leaves	200 mg	247±4.15
12.	Aqueous extract of *Citrus maxima* leaves	300 mg	283.66±5.31^***^

Values are mean±SEM; * *P< *0.05; *** *P< *0.001 vs normal control.

**Table 2.** Effect of aqueous extract of *Citrus maxima* leaves on immobility time in tail suspension test in mice.

Group No.	Treatment	Dose (kg^-1^)	Immobility time (s)
3.	Distilled Water	10 ml	184±7.05
6.	Fluoxetine	20 mg	100.33±5.12***
9.	Imipramine	30 mg	106±6.32***
16.	Aqueous extract of *Citrus maxima* leaves	100 mg	157.5±3.49
17.	Aqueous extract of *Citrus maxima* leaves	200 mg	133.16±4.19*
18.	Aqueous extract of *Citrus maxima* leaves	300 mg	118.5±3.97***

Values are mean±SEM; * *P< *0.05; *** *P< *0.001 vs normal control.

## Discussion

In the present study antidepressant effect of *C. maxima* leaves extract have been studied. The aqueous extract of leaves of this plant showed that it has antidepressant activity. Extract was found to be safe as no mortality was observed following treatment with doses as high as 2000 mg/kg. The increase in locomotor activity indicates a stimulant effect and *C. maxima* has shown stimulant effect in actophotometer. This prompted us to evaluate it further, using paradigms of depression models.

Essential requirement for any antidepressant screening test is prediction about antidepressant activity, with characteristics such as cheapness, robustness, reliability and easy to use (12). Based on these requirements, we selected two behavioral despair models namely TST and modified FST in mice. In the TST, mice are suspended by their tails for a defined period of time and their immobility is assessed. Acute administration of most antidepressants decreases immobility time in TST (13). The immobility exhibited by test animals in these models is indicative of a behavioral despair which reflects a depressive state (6).

In modified FST, when mice are forced to swim in a limited space, they quickly abandon swimming and stand still. Although all antidepressant drugs reduce immobility in the FST, two distinct active behavioral patterns are produced by pharmacologically selective antidepressant drugs (14). Antidepressants that selectively inhibit norepinephrine uptake reduce immobility and selectively increase climbing without affecting swimming. On the other hand serotonin reuptake inhibitors also reduce immobility but increase swimming instead of climbing (15).

The present results showed that the aqueous extract of *C. maxima* was effective in producing significant antidepressant effect in both TST and FST in mice. Aqueous extract of the leaves in all the three doses, were able to reduce immobility and to increase climbing time without significantly affecting swimming time. The effect of high dose of the extract (300 mg/kg) was comparable to standard drug imipramine. The precise mechanisms by which *C. maxima* leaves extract produced antidepressant like effect are not completely understood. However, according to our results, the pattern of behaviors exerted by the extract in the FST is similar to those of imipramine which suggests that this plant extract acts probably by enhancement of norepinephrine neurotransmission as it is related to climbing behavior in the modified FST (14). 

CNS stimulants such as caffeine, theophylline, amphetamine and methylphenidate increases locomotion but they do not possess any antidepressant activity. Tricyclic antidepressants e.g. imipramine have sedative action but fluoxetine (bicyclic) doesn’t possess any sedative action, rather it increased locomotion. The plant extract has shown effect similar to imipramine in modified FST and effect similar to fluoxetine on locomotion; but it’s not necessary that it must possess tricyclic compounds for showing sedative action. It is possible that it may contain organic compound which is not tricyclic one but having potential antidepressant action as well as CNS stimulant action. Therefore we claim that this plant extract possess potential antidepressant activity and it can’t be attributable to its motor-stimulating effects.

## Conclusion

This study showed that aqueous extract of leaves of C*. maxima* possesses antidepressant effects. As the effect of extract was similar to that of imipramine, it may be concluded that this effect might be related to inhibition of norepinephrine uptake which eventually leads to increased availability of norepinephrine in synapses. Further research is underway.
